# Displaced Neer Type IIB distal-third clavicle fractures—Long-term clinical outcome after plate fixation and additional screw augmentation for coracoclavicular instability

**DOI:** 10.1186/s12891-017-1398-3

**Published:** 2017-01-23

**Authors:** Thomas M. Tiefenboeck, Sandra Boesmueller, Harald Binder, Adam Bukaty, Michael M. Tiefenboeck, Julian Joestl, Marcus Hofbauer, Roman C. Ostermann

**Affiliations:** 1 Department of Trauma Surgery, Medical University of Vienna, Waehringerguertel 18-20, 1090 Vienna, Austria; 2 Department of Orthopaedics, Hospital of sacred Heart of Jesus, Vienna, Austria; 3 Division of General Anaesthesia and Intensive Care Medicine, Medical University of Vienna, Vienna, Austria

**Keywords:** Screw fixation, Displaced distal-third clavicle fractures, Retrospective study, Long-term outcome

## Abstract

**Background:**

Unstable Neer Type IIB fractures require meticulous surgical treatment. Thus, the aim of this study was to present long-term outcomes after plate fixation and minimally invasive coracoclavicular (CC) stabilization using screw fixation.

**Methods:**

A consecutive series of patients with unstable Neer Type IIB displaced clavicle fractures, treated by open reduction and internal fixation (ORIF) with a plate and additional screw fixation for coracoclavicular ligament instability, was reviewed in order to determine long-term clinical and radiological outcome.

**Results:**

Seven patients, six males and one female, with a mean age of 37 ± 8 years (median: 36 years; range, 28–51 years), were evaluated. At latest follow-up, after a mean of 67 months (range, 11–117 months), patients presented with the following mean scores: DASH: 0.57, ASES: 98.81, UCLA: 34.29, VAS: 0.43, Simple Shoulder Test: 11.57. However, two complications were observed: one case of implant loosening and one non-union. There were no differences observed between the CC distances comparing postoperative X-rays to those in final follow-up. In 25% of our patients early postoperative complications occurred. In all patients reoperation was necessary to remove the implanted screw.

**Conclusion:**

The results of the present study indicate that the treatment of Neer Type IIB lateral clavicle fractures with ORIF using a plate and additional CC screw fixation, leads to satisfying clinical and radiological outcomes in the long-term. However, considering an early postoperative complication rate of 25% and a 100% rate of secondary surgery due to removal of the CC screw does not seem to justify this technique anymore.

## Background

Fractures of the distal third of the clavicle are comparatively rare, accounting for only 21–28% of all clavicle fractures [1–4]. Due to the rupture of the coracoclavicular (CC) ligaments, Neer Type IIB fractures are defined as unstable fractures which have been shown to exhibit high non-union rates, ranging from 20–44% [5–7], resulting in complications such as persisting pain and limited range of motion (ROM) [8]. Therefore, primary open reduction and internal fixation (ORIF) is usually recommended for these injuries. Surgical treatment, however, is associated with a considerable rate of postoperative complications.

There are various treatment options described in the current literature, however, no consensus is given for the optimal treatment of Neer Type IIB fractures. Several surgical techniques such as tension wire band fixation, use of a clavicle hook plate [9–11], screw fixation [5, 12–15], use of Knowles pins, endobutton fixation, suture anchors and suture tension bands [11, 16–18]—even double plate fixation—are presented. Each of these surgical procedures possesses different limitations regarding complication rates and postoperative function. Furthermore, the vast majority of studies available in the current literature investigated the functional outcome only in the short-term.

Therefore, the aim of this study was to present the clinical and radiological results of Neer Type IIB clavicle fractures treated by ORIF with plate fixation and additional stabilization of the CC ligaments using screw fixation in the long-term.

We hypothesize that Neer Type IIB fractures can be treated by ORIF and CC screw fixation leading to a satisfying long-term outcome.

## Methods

A total of 28 patients with displaced lateral clavicle fractures were treated at this department between March 2003 and February 2010 and were available for retrospective analysis.

Inclusion criteria were as follows: Patients must have (1) suffered a Neer Type IIB clavicle fracture [8], (2) received surgical treatment using plate osteosynthesis and CC screw fixation and (3) been over 18 years of age. Patients with incomplete data sets were excluded from analysis.

The small overall cohort of lateral clavicle fractures (28 patients) finally left seven patients who met the inclusion criteria. All of this can be explained by the sheer nature of this type of injury.

All database files and medical records were reviewed for clinical, functional and demographic data (age, sex, trauma history and treatment modality).

All patients were asked to participate in a clinical and functional examination at latest follow-up, which utilized several outcome scoring systems (Constant Score [19], Disabilities of the Arm Shoulder and Hand (DASH) [20], American Shoulder and Elbow Surgeons Score (ASES) [21], UCLA Shoulder Rating Scale [22] and Simple Shoulder Test [23], as well as the Short Form 36 (SF-36) quality-of-life instrument [24, 25]) . An independent examiner assessed functional and subjective outcome data at routine follow-up. The final follow-up examinations were all performed by the leading author (T.T.). Additional in all patients X-rays were performed at follow-up dates to prove reduction and to grade osteoarthritis according to Kellgren Lawrence Score [26].

**Fig. 1:**
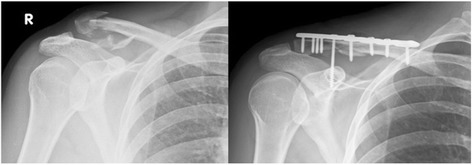
Exemplary case of a lateral clavicle fracture Neer Type IIB treated with a LCP plate and CC screw fixation for coracoclavicular instability

Sports-medicine fellowship-trained shoulder surgeons performed all surgeries under general anesthesia with the patient in beach chair position. A standardized incision of 5–7 cm was made over the lateral clavicle. After exposure, open fracture reduction and fixation via plating was performed. The coracoid process was identified manually and—with a finger on the tip of the coracoid—the clavicle was drilled, aiming the guide wire centrally towards the base of the coracoid process. Direct visualization of the coracoid process was not required in this technique. After length measurement, the guide wire was drilled with a 3.5 mm drill; finally, a screw was placed through a plate hole into the coracoid to fix the CC ligaments. Anatomic reduction was checked via intraoperative fluoroscopy. The delto-trapezoid fascia was reconstructed and wound closure was performed in a standard manner (Fig. 1).

All patients underwent a standardized postoperative rehabilitation program, consisting of compound pendulum exercises following drain removal and first bandage change (on average, on the second postoperative day). Active movement exercises for the elbow joint were allowed directly after surgery. Abduction up to 90° was allowed after stitch removal (on average, 2 weeks after surgery). After screw removal (on average, 8 weeks after implantation), abduction over 90° and full ROM was allowed.

The following postoperative events were defined as major complications, potentially requiring revision surgery: deep surgical site infection, severe postoperative bleeding, nerve palsy, secondary dislocation, screw/plate breakage and bony non-union.

Prior to investigation, ethics approval was obtained by the institutional review board. Informed consent was obtained at follow-up investigation of each patient included.

### Statistical analysis

Descriptive data was reported for the entire patient cohort. Statistical analysis focused on functional and clinical outcomes resulting from the surgical treatment of displaced distal-third clavicle fractures by means of plate fixation and additional screw fixations. Therapeutic variables (surgery, adjuvant therapy and function), pathological variables (re-dislocation rate, limitation of movement) and demographic variables (sex, age and follow-up) were examined.

All calculations were performed using the software suites Microsoft Excel®, SPSS® (Version 22.0, SPSS Inc., Chicago, IL, USA) and GraphPad Prism® (Version 4.00, 2003, GraphPad Software Inc., La Jolla, CA, USA).

## Results

Among all distal-third clavicle fractures treated surgical at this department, we observed an incidence of 25% for Neer Type IIB fractures. The study population consisted of seven patients—six males and one female—with a mean age of 37 ± 8 years (median: 36 years; range: 23–51 years).

A high-energy trauma mechanism was the injury cause in four patients, while a low-energy trauma caused the remaining injuries. The main mechanisms of injury were bicycle and traffic accidents, followed by sport injuries and simple falls. However, in all patients the mechanism of injury was a direct fall onto the shoulder. All patients were treated within a mean of 4 days after injury (median: 2.5 days; range: 1–13 days). The time point of treatment did not influence clinical or functional outcome.

In all patients, the CC screw was removed after a mean time of 2 months postoperatively (median: 1 month; range: 1–2.5 months). In two patients the plate had to be removed as well because of skin irritation.

### Clinical outcomes

At the point of latest follow-up, after a mean of 67 months (range, 11–117 months, median 81.23 months) patients presented with the following mean shoulder scores: DASH: 0.57, ASES: 98.81, UCLA: 34.29, VAS: 0.43, Simple Shoulder Test: 11.57 (Table 1). Based on the evaluated shoulder scores all patients presented with a good functional outcome. Patients also exhibited an overall excellent outcome with regards to the SF-36 at latest follow-up. Only in the sub-scores mental health index and the vitality index the full number of points was not reached, although presenting with a good clinical outcome (Table 2).

**Table 1:** Detailed overview of evaluated scores at final follow-up

Patient No.	Dash	ASES	UCLA	SST	VAS
1	0	100	35	12	0
2	0	100	35	12	0
3	0	100	35	12	0
4	1.5	96.66	33	10	1
5	2.5	94.99	32	11	2
6	0	100	35	12	0
7	0	100	35	12	0
Mean	0.57	98.81	34.29	11.57	0.43
Standard deviation	1.02	2.09	1.25	0.79	0.79

*ASES* American Shoulder and Elbow Surgeons Score, *Dash* The Disabilities of the Arm Shoulder and Hand Score, *No* number, *SST* Simple Shoulder Test, *UCLA* The University of California Los Angeles Shoulder Scale, *VAS* Visual analog Scale

**Table 2:** Detailed SF 36 scores

Case	pfi	rolph	rolem	social	mhi	pain	vital	ghp
1	100	100	100	100	96	100	95	100
2	100	100	100	100	96	100	95	100
3	100	100	100	100	100	100	100	100
4	100	100	100	100	96	100	100	100
5	100	100	100	100	100	100	100	100
6	100	100	100	100	100	100	95	100
7	100	100	100	100	100	100	95	100

*ghp* general health perceptions index, *mhi* mental health index; pain: bodily pain index, *pfi* physical function index, *rolem* role-emotional index, *rolph* role physical index, *social* social functioning index, *vital*: vitality index

### Radiological outcomes

The mean CC distance at the time of injury was 21 mm—compared to 11 mm after surgery and remained unchanged without loss of reduction at 11 mm throughout the final follow-up examination (for detailed data see Table 3). Bony union was achieved in all but one patient. In this patient the non-union was still present 11 months after primary surgery, however, this patient presented with good final results at latest follow-up.

**Table 3:** Detailed CC distances in mm during follow-up

Patient No.	CC distance pre operative in mm	CC distance post operative in mm	CC distance after screw removal in mm	CC distance at final follow-up in mm
1	20	10	9	10
2	21	10	10	10
3	22	11	10	10
4	26	14	14	14
5	20	10	10	10
6	20	10	10	10
7	18	11	11	11
Mean	21.00	10.86	10.57	10.71
Standard deviation	2.52	1.46	1.62	1.50

*No*. number, *CC* coracoclavicular, *mm* millimeters

In two patients signs of mild osteoarthritis according to Kellgren Lawrence [26] were found at latest follow-up in the other patients no signs of osteoarthritis were found. Prior to surgery in our patient collective no signs of osteoarthritis were seen.

### Complications

Overall, complications occurred in two patients during the follow-up period—one non-union and one case of implant loosening—requiring surgical intervention in the latter. This patient’s loosened implant (the culprit being the screw fixing the CC ligaments) was simply removed, and the patient presented free of any complications at the latest follow-up.

## Discussion

The most important finding of the present study was that plate fixation and minimally invasive CC stabilization presents with excellent outcome regardless of postoperative complications at long-term. Our patients completed the study with the following mean scores: DASH: 0.57, ASES: 98.81, UCLA: 34.29, VAS: 0.43, Simple Shoulder Test: 11.57. Patients also exhibited an overall excellent outcome with regards to the SF-36. There were no signs of recurrent instability at the time of latest follow-up. The final mean measured CC distance was 10.5 mm ± 1.62 mm, which is well in line with data presented by Hermann et al. [27].

However, the combination of plate fixation and CC screw fixation revealed a relatively high complication rate of 25%—which has to be considered when comparing various surgical procedures. Nevertheless, this complication rate is well in line with the current literature [28] pertaining to this technique. Furthermore, it has to be considered that there is a 100% rate of secondary surgical intervention to remove the CC screw after healing of the CC ligaments, in contrast to other newer techniques like endobutton fixation, which is a definitive surgical technique. Therefore, it has to be questioned if a surgical procedure with a 100% reoperation rate is still adequate.

Unstable distal clavicle fractures (i.e., Neer Type IIB) are prone to delayed union or non-union, recurrent pain and instability caused by significant fragment dislocation. In most of the cases, the lateral fragment is small and of poor bone quality; therefore, vertical stability cannot be sufficiently achieved with plate fixation alone.

Several different techniques for the reconstruction of unstable distal-third clavicle fractures have been presented in literature [28–30]. Specifically concerning the reconstruction of the CC ligaments, several minimally invasive devices are recommended. The current literature yields only a handful of studies dealing with this special type of fracture. However, these studies reported achieving overall good-to-excellent results [6, 16, 29–31], which is in accordance to the findings of this study. Interestingly, a recent publication by Shin S et al. presented plating alone without CC screw augmentation to be a safe procedure without loss of reduction [32].

In the literature, the use of additional screw fixations is associated with a high rate of complications, such as migration, infection, breakage, and loosening of the implant [12]. In this series, we observed complications in two patients—one case of implant loosening and one non-union. However, an overall complication rate of 25% is nonetheless high and needs to be considered when choosing this technique for reconstruction.

Several studies have revealed a correlation between fracture displacement and worse outcome. This fracture feature (i.e., displacement) is associated with high-energy trauma [33–35]—an association we were able to confirm in our series.

Our results suggest that an immediate surgical treatment of Neer Type IIB fractures, with an additional use of screw fixation to reconstruct the CC ligaments, leads to good long-term radiological and functional outcomes. However, more accurate methods than additional screw fixation should be considered for CC reconstruction, in order to avoid re-operation for implant removal and even possibly the high rates of early postoperative complications as presented in our study and in the literature [9, 29–31].

This study does possess considerable limitations, mainly seen in its retrospective design, small study cohort and the vast time-span of treatment—though all of this can be explained by the sheer nature of this type of injury. In general, the incidence of unstable lateral clavicle fractures is low, and our analysis only focused on Neer Type IIB fractures. The number of patients included in our study is low, however, comparable to other studies presented in the literature [27]. Nevertheless, to the best of our knowledge, this is the first investigation analysing the long-term outcome after surgical treatment of Neer Type IIB fractures. Future prospective, multi-center studies with long-term follow-up schedules will help to determine the most appropriate surgical method for treating this complex fracture type.

## Conclusion

Our results indicate that the treatment of Neer Type IIB lateral clavicle fractures with open reduction, plate fixation and additional CC screw fixation leads to satisfying clinical and radiological outcomes in the long-term. However, an early postoperative complication rate of 25% and the need of re-operation in all patients to remove the CC screw lead to the assumption that this technique should not be recommended anymore.

## Abbreviations

AC: Acromioclavicular
ASES: American Shoulder and Elbow Surgeons
CC: Coracoclavicular
DASH: Disability of the arm, shoulder and hand score
ORIF: Open reduction and internal fixation
ROM: Range of motion
UCLA: University of California at Los Angeles Shoulder Score
VAS: Visual analog scale
